# Timelier notification and action with mobile phones–towards malaria elimination in South Africa

**DOI:** 10.1186/1475-2875-13-151

**Published:** 2014-04-21

**Authors:** Vanessa Quan, Anette Hulth, Gerdalize Kok, Lucille Blumberg

**Affiliations:** 1 Division of Public Health Surveillance and Response, National Institute for Communicable Diseases (NICD) of the National Health Laboratory Service, Private Bag X4, Sandringham 2131, Gauteng, South Africa; 2 School of Pathology, University of the Witwatersrand, Johannesburg, South Africa; 3 Department of Public Health Sciences, Karolinska Institutet, Stockholm, Sweden; 4 Malaria Control Programme, Mpumalanga, South Africa

**Keywords:** Malaria surveillance, Elimination, Evaluation, Mobile phone, SMS notification, South Africa, Rural

## Abstract

**Background:**

Surveillance with timely follow-up of diagnosed cases is a key component of the malaria elimination strategy in South Africa. The strategy requires each malaria case to be reported within 24 hours, and a case should be followed up within 48 hours. However, reporting delays are common in rural parts of the country.

**Methods:**

A technical framework was implemented and for eight months a nurse was hired to use a smartphone to report malaria cases to the provincial malaria control programme, from selected primary health care clinics in a rural, malaria-endemic area in South Africa. In addition, a short text message (SMS) notification was sent to the local malaria case investigator for each positive case. The objective was to assess whether reporting over the smartphone led to timelier notification and follow-up of the cases. An evaluation on the simplicity, flexibility, stability, acceptability, and usability of the framework was conducted.

**Results:**

Using mobile reporting, 18 of 23 cases had basic information entered into the provincial malaria information system within 24 hours. For the study period, the complete case information was entered two to three weeks earlier with the mobile reporting than from other clinics. A major improvement was seen in the number of positive cases being followed up within 48 hours. In 2011/2012, only one case out of 22 reported from the same study clinics was followed up within this timeframe. During the study period in 2012/2013, 15 cases out of 23 were followed up within two days. For the other clinics in the area, only a small improvement was seen between the two periods, in the proportion of cases that was followed up within 48 hours.

**Conclusions:**

SMS notification for each diagnosed malaria case improved the timeliness of data transmission, was acceptable to users and was technically feasible in this rural area. For the malaria case investigations, time to follow-up improved compared to other clinics. Although malaria case numbers in the study were small, the results of the qualitative and quantitative evaluations are convincing and consideration should be given to larger-scale use within the national malaria control programme.

## Background

South Africa’s successful malaria control programme, which included the introduction of artemisinin-containing combination treatment (ACT), an effective residual indoor insecticide spraying programme, together with the adoption of regional malaria control strategies in South Africa, Mozambique and Swaziland, produced a decline in malaria case numbers nationally, from 26,506 in 2001 to less than 10,000 by 2011 [1]. According to the malaria pre-elimination phase criteria set out by the World Health Organization of five cases per 1,000 population at risk, South Africa has been earmarked to achieve malaria elimination [2]. South Africa is one of 34 malaria-endemic countries currently targeting elimination of the disease (that is, no local malaria transmission in a defined geographical area), with the goal to achieve elimination by 2018 [3]. Active surveillance is a key component of the elimination strategy in order to break the chain of transmission, through timely diagnosis and treatment of parasitaemic, but often asymptomatic, household or community contacts of a confirmed malaria case. This is because the reduction in numbers of infected people shrinks the reservoir of malaria parasites available for transmission. The monitoring and evaluation target indicators include, amongst others, notification of all malaria cases within 24 hours of diagnosis at all public and private health care facilities, investigation within a case’s household and neighbouring households within 48 hours of notification, and reporting of each case to provincial and national level within 72 hours of notification [4].

**Figure 1:**
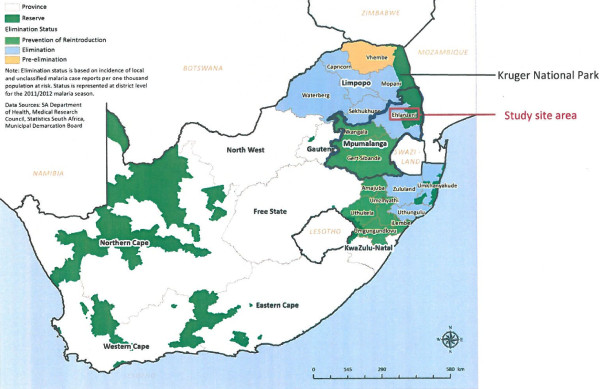
**Study site area in Mpumalanga Province on map of South African malaria districts elimination status, 2011–2012 **[5]**.**

Despite a decreasing number of malaria cases, response to notification and follow-up of each case is extremely important to break the chain of transmission through treatment of identified parasitaemic persons, and reach the target for elimination. Mpumalanga Province, which is one of three malaria-endemic provinces in South Africa, includes the southern region of the Kruger National Park and borders on Mozambique (Figure 1). In the 2012/2013 season, there were 3,007 cases reported from Mpumalanga, out of which 273 were a result of local transmission. Of all cases, 16 died from their malaria infection (personal communication with Provincial Malaria Programme).

The vertical national malaria programme depends on the provincial malaria programmes for malaria control field activities and collection of data on confirmed cases. Malaria cases are usually managed in rural, primary health clinics where the diagnosis is based on the use of rapid diagnostic tests, and immediate treatment is provided. Staff at health facilities complete a paper-based malaria notification form when there is a positive malaria case (a symptomatic person with a positive rapid malaria diagnostic test). This form is collected by a malaria case investigator who visits a number of health facilities once or twice a week. From the address information on the notification form, the malaria case investigator visits each case and the immediate local community. The form is taken to a different subdistrict for entry of the information into a malaria information system for the province. Therefore, with current routines, it may take a long time before the notification form is collected by the malaria case investigator, and much longer before the information is entered into the provincial malaria information system.

The logistical challenges are numerous and include shortage of case investigators, transport problems, insufficient funding to support phone calls, petrol and car maintenance, and case patients being absent from home when the malaria case investigator visits. The infrastructure is poor, in that facilities do not have fax machines or email facilities to send urgent notifications to the malaria programme, and electricity supplies may be intermittent. The whole process has been found to be slow and inefficient, impacting negatively on the malaria case notification and investigation time targets.

There are many reports of mHealth (mobile technologies for health) as an innovative tool for improving health care quality in middle and low income countries [6,7]. Mobile technologies have been used for faster reporting of information, with usually a higher cost for initial set-up but a long-term overall cost-effective benefit. The evidence to date is that mobile electronic technologies are useful in terms of quality, timeliness and overall cost-effectiveness for surveillance reporting and research studies, and that mobile technologies are acceptable and feasible in lower resource settings [8,9]. South Africa is amongst countries with the highest proportion of mobile phone users per population: 93% of the population subscribe to a mobile phone network [10]. Only five million of South Africa’s estimated 50 million population use landline phones. Text messaging via SMS is the most common function among South African mobile customers, and is used by almost 4.2 times more people than email. More than two-thirds (69%) of consumers prefer sending texts to calling, largely because it is less expensive, and 10% of people believe texting to be a more rapid way of communicating [11].

This article describes a pilot study of mobile phone reporting of malaria cases diagnosed at rural primary health care clinics in the Mpumalanga Province of South Africa. The main purpose of the study was to assess how mobile reporting of malaria cases could assist in meeting the operational requirements of malaria notification and case follow–up, as well as to test the technology in a rural setting in the context of a functioning malaria control programme.

## Methods

In order to establish whether mobile phone reporting could assist in all malaria cases being notified within the required time frame, a mobile phone technical framework was implemented and a nurse was hired to send reports over a smartphone from selected primary health care clinics in a malaria-endemic area in South Africa for eight months. The work was done in close collaboration with the Mpumalanga Malaria Control Programme, which normally receives the paper-based reports from the clinics included in the study. The mobile reporting did *not* replace the submission of the complete notification forms during this study. As timely reporting is valuable only if action is taken, an evaluation on whether reporting over the mobile phone led to timelier follow-up of the cases was also conducted.

Ethical approval was obtained from the University of the Witwatersrand Human Research Ethics Committee (protocol number M120666) and the Mpumalanga Provincial Government.

### Technical set-up

A mobile form collection and server solution was implemented, making use of open-source software configured to fit the specific use-case and requirements of the study. ODK Collect [12] was used for mobile data entry, which runs as a Java-based application on an Android smartphone. The software allows for data to be stored on the mobile and sent at a later stage, should networks be temporarily unavailable. Mobile forms were authored using the XLSForm standard and form submissions were maintained using a custom implementation of Formhub [13] running on a dedicated server hosted in South Africa. The reason why a dedicated server was set up for the study, was to not store patient-level data outside of South Africa (in the so called cloud). The Formhub web interface was only accessible over a secure connection using Secure Sockets Layer (SSL) with mobile form submissions secured in the same way. Access to form data and data export functionality was controlled on a per-user level, through appropriate authentication and authorization mechanisms configured in the Formhub implementation.

### Data collection

Patient-level data on each malaria case, as currently reported within the malaria control programme, were submitted to the central server on a form on the smartphone that had the same structure and fields as the paper malaria case notification form.

In this study a designated person reported from the health care facilities, as the regular staff, with their current staff complement and work descriptions, were not able to handle this kind of reporting. To make the reporting as authentic as possible, a nurse was hired for the task. This person was elderly (71 years), with very little experience of computers and no experience of using smartphones. She contacted the clinics each weekday to inquire about any new malaria case, in order to conform to the 24-hour reporting limit. If a case had been found, the nurse would travel to the clinic and then send the required information over the phone to the server. She was asked to keep a manual log of all irregularities during the whole study period.

As the chosen technical solution lacked a function for automatic notification of new data having been submitted, it was decided at the beginning of the study that the nurse should send a text message via SMS to the malaria team coordinator in the district, for each new submission that was made. The malaria team coordinator, in turn, forwarded this message to the local case investigator. This message comprised the name of the clinic, the patient’s name and age, and date of malaria diagnosis. This basic information was entered into the malaria surveillance information system in the province upon reception.

### Study site

The reporting was done from three primary health care facilities in a rural, resource-limited area of Mpumalanga Province with all the challenges regarding communications (fax and conventional phone often do not work), supply of electricity and transportation. The mobile phone reception in this area is good and many more people rely on mobile phones than on conventional land lines.

### Study period

The reporting was done from 1 October, 2012 to 31 May, 2013 (eight months) to cover the usual malaria season in southern Africa.

### Evaluation

The following measures were evaluated using a mixed methods approach: simplicity, flexibility, stability, timeliness, and acceptability [14]. The usability of the framework, ie, how easy it was to use the device and the software was also evaluated.

### Qualitative evaluation

A structured interview was conducted with all individuals who were involved in the mobile reporting, from the health care workers at the clinics and the nurse doing the reporting to the stakeholders at the district communicable diseases control office (n = 9). The evaluation also included an analysis of the manual logs that the nurse kept and a record on how often the phone had to be charged and how many technical troubles were encountered (‘battery and phone trouble study’). The results of the interviews formed the basis for the evaluation of the simplicity, flexibility and stability of the software and mobile device. Based on these interviews, the usability and acceptability of the surveillance system was also evaluated.

### Quantitative evaluation

The timeliness was evaluated with respect to two events:

1) Notification: whether the time from diagnosis to entering the case information into the provincial malaria information system – thus making the information available to relevant public health authorities – was affected with mobile reporting in place. The time it took for the basic information sent via SMS to be entered, as well as the time between diagnosis and the complete information being entered were evaluated.

2) Follow-up: whether the time it took from diagnosis to case follow-up was affected by the mobile reporting. The number of days it took for the cases to be followed up after diagnosis was measured. The proportion of cases that were followed up within the required time (48 hours), that is, within zero, one or two days from notification was calculated. Only access to dates and not the hours was available, and therefore an assumption was made that if a case was followed up within two days, this complied with the 48 hours’ recommendation. In practice this may correspond to some additional hours. The notification date was assumed to be equal to diagnosis date, as a nurse would fill out a form immediately upon diagnosis.

The two indicators were investigated for the three clinics as well as for other clinics served by the same malaria case investigator for the study period. To control for other factors that might have affected the reporting, the difference in timeliness between the three study clinics to other clinics served by the same case investigator for the previous year when no study was conducted, was also compared. Additionally, the timeliness for all other clinics in the municipality, to use as an unbiased control, for both time periods was computed.

To enable these comparisons the following attributes for each case were collected:

• Date of diagnosis.

• Date of receipt of notification SMS.

• Date of entry of paper-based form data into the provincial malaria information system.

• Date of case follow-up.

An exploration into whether the district malaria control programme had received any paper forms from the three clinics without any reports being sent by the mobile device was performed.

Baseline data for all clinics in the area for the study period and the control period were provided by the Mpumalanga Malaria Control Programme in Mbombela.

## Results

### Simplicity, flexibility and stability

ODK Collect, which was used for mobile data entry, was deemed simple and intuitive to use; it had a limited set of functionality and a low-learning curve. Asynchronous form submission added flexibility by allowing for data to be collected without a connection, and submitted at a later stage. There were, however, some issues with stability when using an older smartphone during the time when the study was set up. These issues were resolved when upgrading to the newer phone, which was then used for the study period. The back-end server database (Formhub) was simple to set up and use. Some technical knowledge was required to design the forms, and to manage and maintain the system. To set up a custom-hosted instance was, however, not as simple. Documentation and support for hosting a separate instance of Formhub was limited. Once all issues were resolved, the system proved to be stable. The mobile application used (ODK Collect) required an Android-based smartphone. This hardware requirement was suitable for this specific study, but may not be viable for any broader roll-out.

The results of the ‘battery and phone trouble’ study showed that there were no regular problems with low battery. It was impossible to send data from one of the three clinics during the study period because of poor connectivity. Instead, the nurse sent the forms when she was on the road home as the data could be collected offline, stored on the phone and sent by bulk later if connection was unavailable. The notifications sent via SMS worked well from all three clinics. There was a technical failure of the system with receiving the forms at the central server for a period of six weeks, but the SMS notifications were still being sent and received during that period.

### Usability

The nurse who submitted the reports had never used a smartphone before entering the study. She had used a computer for 1.5 months in 2006. Nonetheless, two hours of training were sufficient, and after three weeks she felt comfortable with the forms and the device. The foremost difficulty was to accurately handle the touch screen, as she was unused to touch screen devices. The size of the screen was perceived as adequate, and the size of the device was deemed good as it was easy to store.

Once the nurse was used to the mobile phone it took only three minutes to complete the malaria form. All data were entered from the paper-based forms. Having all information on paper substantially increased the nurse’s confidence. It should be noted that the paper-based form was more quickly filled out than the equivalent form on the smartphone.

### Acceptability

The staff at the clinics perceived that the study made their work on reporting infectious diseases more meaningful as it was evident that someone actually reviewed the data they were collecting and submitting. The study also increased the understanding of the importance of early reporting, according to the nurses at the health clinics.

One of the interviewed nurses stated that ”it will not be a problem if the malaria SMS alert was left up to the clinic because it would be easy and the benefits are good”. All three clinics were willing to continue with mobile reporting by, for example, sending information on malaria because they see the benefit of early reporting and case finding.

### Timeliness

In Table 1, the results for the time from case diagnosis to entry of case information into the provincial malaria information system are presented. Data are shown for three groups of clinics: the three clinics included in the study, other clinics served by the same malaria case investigator, and, all other clinics in the municipality. Data are presented for two time periods: the study period and the same period for the previous season. Table 2 contains data on timeliness from diagnosis to follow-up, for the same three groups of clinics and the same two time periods as in Table 1.

**Table 1:** Time between diagnosis and case information being entered into the provincial malaria information system

	**Clinics with mobile reporting**		**Other clinics with same case investigator as those with mobile reporting**		**Other clinics in the municipality with other case investigators**	
**Time period**	October 2011-May 2012	October 2012-May 2013	October 2011-May 2012	October 2012-May 2013	October 2011-March 2012	October 2012-March 2013
**# of clinics**	3	3	11	16	27	41
**# of reported cases**	22	23	42	66	168	170
**# of days on average (range)**	33.0 (12–60)	7.3 (2–19)	35.2 (7–78)	28.9 (7–87)	33.4 (8–79)	42.6 (6–187)
**Median # of days**	37	7	27	22	29	27

**Table 2:** Time between diagnosis and follow-up

	**Clinics with mobile reporting**		**Other clinics with same case investigator as those with mobile reporting**		**Other clinics in the municipality with other case investigators**	
**Time period**	October 2011-May 2012	October 2012-May 2013	October 2011-May 2012	October 2012-May 2013	October 2011-March 2012	October 2012-March 2013
**# of clinics**	3	3	11	16	27	41
**# of reported cases**	22	23	42	66	168	170
**# of days on average (range)**	14.7 (0–40)	2.5 (0–10)	18.5 (0–65)	5.3 (0–32)	11.8 (0–69)	23.5 (0–180)
**Median # of days**	13.5	2	8	4	6	4
**# of cases followed up within 2 days (% of total # of reported cases from the covered clinics)**	1 (5%)	15 (65%)	5 (12%)	22 (33%)	22 (13%)	62 (36%)

During the study period, 24 cases of malaria were reported from the three clinics from which case information and the SMS notification was sent by mobile phone. For one case, relevant data from the provincial malaria information system were not available to include in the evaluation, which hence encompassed 23 cases. All positive cases that were notified from the three clinics were also notified by mobile phone.

Data were incomplete for April and May 2012 for the clinics in the municipality served by other case investigators. For this reason these two months were removed from the 2013 data on clinics served by other case investigators, to make the numbers comparable. However these two months were kept in the datasets for the study case investigator analysis, as the number of reported cases was already low. What was important in the analysis was the proportions rather than the absolute numbers, and the figures are thus still comparable between the two time periods for the three groups of clinics in the two tables. In addition, April and May were two months with a rather low number of reported malaria cases.

During the study period, for the 23 cases from the three clinics included in the study, the basic information contained within the text message was entered into the provincial malaria information system in less than or equal to one day. The exceptions were when the diagnosis was made on a Friday and the reporting nurse was at another clinic, or when the diagnosis was made on Saturdays or public holidays. The average time between reporting and data being entered was one day (range, zero to five days), and the median was also one day. Eighteen cases out of 23 (75%) had the basic information entered within 24 hours.

The median number of days between diagnosis and the complete case information being entered into the malaria information system for the province, changed from 37 days in 2011/2012 to seven days during the study period for the three clinics (Table 1). Comparing to the other clinics with the same case investigator, the number of days decreased from 27 in 2011/2012 to 22 in 2012/2013. For clinics in the municipality with other case investigators, there was also a small improvement, namely, two days, between 2011/2012 and 2012/2013, meaning that the whole improvement of 30 days cannot be attributed to the mobile-based reporting. Nonetheless, with the case investigator being notified about positive cases, the reporting was two to three weeks faster than from other clinics in the municipality.

An improvement was seen in the number of malaria cases that were followed up within the required 48 hours (Table 2). In 2011/2012 only one case out of 22 (5%) reported from the three clinics in the study was followed up within this time. During the study period in 2012/2013, 15 cases out of 23 (65%) were followed up within two days. An improvement between the two seasons in the proportion of cases that were followed up within 48 hours could also be noted for the other clinics in the municipality: 33% in 2012/2013 compared to 12% in 2011/2012 for the clinics with the same case investigator and 36% in 2012/2013 compared to 13% in 2011/2012 for the clinics with other malaria case investigators.

## Discussion

The selected technical solution was deemed adequate for the study. Overall, it was particularly the notification sent via SMS by the reporting nurse to the malaria team coordinator that had the largest impact on the positive outcome of the study. SMS reporting of weekly aggregated malaria data from health facilities supports malaria control programmes in Zanzibar and Zambia [15,16].

One limitation of this study is the small number of malaria cases covered. This is largely because of the successes of the malaria control programme in South Africa. Despite the small numbers, every case is important and requires timely notification and response, and this will become increasingly so as the target date (2018) for malaria elimination in South Africa approaches.

The time it takes between diagnosing a case and for the case information to be notified, and thus making the information available for all relevant stakeholders, should preferably be 24 hours or less. As seen in the results (presented in Table 1), no single report from this area complied with this requirement. The reason for this is that the paper reports need to be fetched by car and driven to the city where the entries are being made. A case will be followed up regardless of whether it is entered into the provincial malaria information system. It is, however, important for disease monitoring that cases are captured in a timely manner, so that all stakeholders have access to the same information. Within the study, the data capturers were not given access to the database to which the reporting nurse submitted the data. With the reporting on a smartphone, the same information was entered as on the paper form, and the study showed that data capturing within 24 hours is feasible from a technological point of view. However, an important aspect is that there was a designated person doing the data entering on the phone. At the present time it is difficult to foresee a countrywide system requiring all malaria case information to be entered via a smartphone. Should this eventually be done, the manual SMS should be replaced by an automatic notification for all relevant recipients.

The timeliness for both data capturing and follow-up improved between the 2011/2012 and the 2012/2013 seasons. One reason could be that not only the case investigator serving the clinics from which the mobile reporting was done was affected by the study’s presence, but that the study had an impact on the entire case investigator team. Other reasons could be that the weather conditions were more challenging during the 2011/2012 season. However, as the improvement was much larger for the three study clinics than for the other clinics served by the same case investigator, the contribution of the mobile reporting can be quantified.

It was also noted by the nurses at the participating clinics that with the SMS alerts, the case investigator came immediately for positive cases: “the malaria team came 20 times quicker than normal”. By notifying the case investigator of each new case, transport costs were also reduced as the case investigator would only travel to a clinic when there was a case to report and follow up.

In this study, a designated person sending the reports was employed which made it feasible to evaluate the usage of a smartphone. The phone was found both useful and acceptable, but it would not be possible to have the nurses with their current staff complement and work descriptions to handle any kind of reporting requiring smart phones and specific applications.

Furthermore, the more advanced system of transferring the data to a central database is not yet applicable in this setting and therefore on a larger scale it would not be possible at present. Leon and Schneider mention in detail the challenges of setting up a large scale mHealth in South Africa, including a weak health system, organizational weaknesses and a gap between policy and implementation (which includes the introduction and use of information technology) and poor capacity of provinces and districts to use information systems [10] .

A more viable way could be to introduce a toll-free number in the country to which any nurse who has filled out a malaria notification form could send an SMS with some basic information, such as name of the clinic, name and age of the case, and the date of diagnosis, to a central coordinating hub. Such a system would require that the toll-free number be stated on the malaria form and that the information is distributed from the central hub to the district coordinator, who can then mobilize the investigating team. Should such a system be implemented, it would be of great value for the central data collection if this information, contained within the SMS, could be automatically integrated into the various provincial information systems for case-based data on malaria.

## Conclusion

The presented study showed that mobile phone-based reporting of notifiable diseases was acceptable to the users and technically feasible in a rural area in South Africa. A retired nurse without computer skills felt comfortable using a smart phone for reporting after three weeks, after having received only two hours of training. The study also had a positive effect on the nurses at the clinics, as they realized that the reporting of notifiable diseases is actually recognized by the authorities and has a clear purpose.

The use of a notification sent via SMS from the mobile phone for each newly diagnosed malaria case improved the timeliness. Relative to other clinics and previous season, the time between diagnosis and reporting decreased substantially as did the time between diagnosis and follow-up. Notably, the median number of days between diagnosis and follow-up for the three study clinics decreased to two days, which is the target set within the malaria elimination strategy in South Africa.

A vision is that full malaria case reporting could be done in real-time over a smartphone by the health care professionals at the primary health clinics in the future. However, smartphones may be useful for research projects and dedicated surveillance programmes, but presently cannot be used on a more general, national level in South Africa. For this to happen, the reporting system would need restructuring over the whole spectrum of notifiable diseases.

Although malaria case numbers were small in the presented study, the results are convincing. It is a proof of concept that a complementary SMS sent by the reporting nurse can be an important component to reach the goal of having no local malaria transmissions by 2018. Consideration should be given to large-scale use, possibly using a toll-free phone service, within the provincial malaria control programmes.

## Competing interests

The authors declare that they have no competing interests.

## Authors’ contributions

LB, AH and GK conceptualized the study; VQ coordinated the study and drafted the manuscript; AH did the analyses and drafted the manuscript; GK informed on malaria programme requirements and operational outcomes of the study. All authors read and approved the final manuscript.
